# A Large Extension to HIV-1 Gag, Like Pol, Has Negative Impacts on Virion Assembly

**DOI:** 10.1371/journal.pone.0047828

**Published:** 2012-10-23

**Authors:** Hiyori Haraguchi, Takeshi Noda, Yoshihiro Kawaoka, Yuko Morikawa

**Affiliations:** 1 Kitasato Institute for Life Sciences, Kitasato University, Minato-ku, Tokyo, Japan; 2 Institute of Medical Science, University of Tokyo, Minato-ku, Tokyo, Japan; 3 ERATO Infection-Induced Host Responses Project, Japan Science and Technology Agency, Saitama, Japan; 4 Influenza Research Institute, Department of Pathological Sciences, University of Wisconsin-Madison, Madison, Wisconsin, United States of America; Queensland Institute of Medical Research, Australia

## Abstract

The GagPol protein of HIV-1 harbors viral enzymes, such as protease (PR), reverse transcriptase, and integrase, that are all crucial for virion infectivity. Previous studies have suggested that expression of GagPol alone does not produce viral particles and that the budding defect is caused by the presence of the Pol region. However, it has remained unknown why GagPol fails to produce viral particles. We show here that HIV-1 GagPol is incapable of membrane binding and subsequent particle assembly. Our confocal data indicated that, despite full N-myristoylation, GagPol protein failed to target plasma membrane with diffuse distribution in the cytoplasm. Membrane flotation analysis confirmed these findings. Progressive C-terminal truncation of GagPol to give GagPR allowed for plasma membrane targeting but still not for particle production. Conversely, the C-terminal addition of a noncognate protein, such as ß-galactosidase or 4 tandem GFP, to Gag impaired the membrane affinity, indicating that the Pol region, a large extension to Gag, inhibits membrane binding in the context of GagPol. The addition of the 10 N-terminal amino acids of Fyn kinase [Fyn(10)], a tight membrane-binding signal, conferred plasma membrane targeting on GagPol, but the Fyn(10)GagPol did not produce viral particles. The defect in particle budding was not rescued by the introduction of the PTAP motif, which is responsible for a late stage of viral particle budding. Rather, electron microscopy suggested that the budding defect of GagPR occurred at an early stage of particle morphogenesis. Our data, which were consistent with previous observations, demonstrate the defects of GagPol in membrane binding and particle assembly.

## Introduction

The human immunodeficiency virus type 1 (HIV-1) genome contains three major genes, *gag, pol*, and *env*, that encode the viral structural protein Gag, enzymatic polyprotein Pol, and envelope protein Env, respectively. The *gag* gene is translated into a Gag precursor protein that is subsequently cleaved into the p17/matrix (MA), p24/capsid (CA), p7/nucleocapsid (NC), and p6 domains by HIV-1 protease during virion maturation. The *pol* gene is translated into a GagPol precursor protein by −1 ribosomal frameshifting, which occurs at an efficiency of 5–10% during Gag synthesis, resulting in the generation of a 10–20∶1 ratio of Gag to GagPol [1], [2]. GagPol is essential for viral replication, since the Pol region harbors viral-specific enzymes [protease (PR), reverse transcriptase (RT), and integrase (IN)] that are indispensable for virion infectivity.

The Gag protein is a driving force for retroviral particle assembly. This process consists of several distinct but mutually interdependent steps, including the membrane targeting and multimerization of Gag as well as the pinching off of budded particles from the membrane. In HIV-1, Gag multimerization is driven by the CA to NC domain [3]–[7]. The membrane-binding domain for HIV-1 Gag is composed of an N-terminal myristoylation signal [8], [9] and a cluster of basic residues in MA [10], both of which are required for tight membrane binding of Gag [10]. Nuclear magnetic resonance (NMR) studies have suggested a myristoyl switch model in which the N-myristoyl moiety is exposed upon the binding of phosphatidylinositol 4,5-bisphosphate [PI(4,5)P_2_] to the basic residues [11]. Although the N-myristoyl moiety is not directly involved in Gag multimerization, several studies have suggested that myristoyl exposure is regulated by Gag multimerization [12]–[15]. HIV-1 particle budding requires the sequential recruitment of the host endosomal protein sorting complex required for transport (ESCRT) components to the site of particle assembly [16], [17]. Like other retroviruses, the p6 domain contains the late domain (the PTAP motif) that interacts with the ESCRT components [16], [18]–[20]. A number of studies indicate that HIV-1 Gag primarily targets the plasma membrane, where particle assembly and budding occur [21]–[26], although Gag also can initiate assembly in endosomes and then be transported to the cell surface [27]–[34].

In contrast, GagPol itself lacks the ability to produce viral particles and incorporates into viral particles only through coassembly with Gag [35]–[39]. The N-terminal region of GagPol is identical with the major part of Gag (MA-CA-NC). However, GagPol lacks the p6 domain and instead contains the p6* domain, which is a linker region between NC and PR and lacks the PTAP motif. The defect of GagPol in particle assembly, when the GagPol contains active PR, is partly ascribed to premature processing before particle assembly by the overexpression of PR, since the overexpression of GagPol and that of the active PR dimer have been shown to result in no particle production [40]–[44]. While the treatment with PR inhibitors partially suppressed this defect, GagPol remained very inefficient at particle production [42], suggesting that, even when it contains inactive PR, GagPol is incapable of particle production. A very recent study investigated this possibility, revealing that the Pol region but not the p6 domain was responsible for the budding defect of GagPol [45]. However, it remains unanswered why GagPol protein is incapable of particle production. Specific questions should address at which stages and by what mechanisms the Pol region imposes the defects.

One of the difficulties in investigating GagPol trafficking is the low level of GagPol expression relative to that of Gag. Moreover, the N-terminal half of GagPol is identical to Gag, and this hinders discrimination between the two. To overcome these difficulties, we previously generated the HIV-1 molecular clone derivative containing two distinct epitope tags [FLAG and hemagglutinin (HA)] to the C-termini of Gag and GagPol, respectively [44]. The data from that study showed that both GagPol and Gag, when coexpressed, were relocated from the cytoplasm to the plasma membrane and were incorporated into viral particles, the yield of which was equivalent to that of the wild type of the HIV-1 molecular clone [44]. Using our GagPol constructs, we here address the budding defect of GagPol. Our data indicate that GagPol is originally incapable of membrane binding and that plasma membrane targeting, even when conferred, is insufficient for particle production, suggesting that other stages (e.g., multimerization) are also potentially impaired by the Pol region.

## Materials and Methods

### Construction of HIV-1 Molecular Clones

The derivative of HIV-1 molecular clone pNL43 containing inactive PR [46] was used as the wild type (WT) in this study. The pNL43 derivatives, Gag-FLAG/Pol-HA and GagPol-HA, both of which contain inactive PR, were described previously [44]. Briefly, the Gag-FLAG/Pol-HA expresses Gag with a C-terminal FLAG sequence and GagPol with a C-terminal HA sequence. The GagPol-HA contains a deletion of the frameshifting signal and expresses GagPol with a C-terminal HA sequence but does not express Gag. The pNL43 derivative expressing Gag-FLAG without GagPol was also described previously [44]. The pNL43 derivatives expressing truncated GagPol proteins were generated by the insertion of a premature termination codon at the junction of PR-RT and RT-IN. For the replacement of the Pol region with ß-galactosidase (ß-gal) or green fluorescent protein (GFP) in the context of the GagPol protein, unique NotI and XbaI sites were initially created at the p6*-PR junction using 5′-TTCGCGGCCGCTGCTTCTAGACCTCAGATCACTCTTTGGCAGCGA-3′ and 5′-AGGTCTAGAAGCAGCGGCCGCGAAGCTAAAGGATACAGTTCCTTGT-3′ (underlined, NotI and XbaI linkers), and the ß-gal or GFP gene containing the termination codon was cloned in-frame into the NotI-XbaI junction. To generate the pNL43 derivative expressing the Gag-4GFP construct, four GFP fragments were ligated at each end using restriction sites (XbaI-BamHI-KpnI-SacI-EcoRI) and were fused with the C-terminus of Gag. GagPol constructs containing the p6 domain instead of the p6* domain were generated by the insertion of four nucleotides into the p6/p6*-PR junction (nucleotide positions 2249–2252), which placed the *gag* and *pol* genes in the same reading frame at the p6-PR junction. The C-terminal truncation of these GagPol constructs was similarly carried out by inserting a premature termination codon at the PR-RT junction. The pNL43 derivatives containing the 10 N-terminal amino acids of Fyn kinase [Fyn(10)] at the N-terminus of Gag were constructed from pNL43/Fyn(10)fullMA as described previously [47]. To create the PTAP motif within the p6* domain, the 12 nucleotides corresponding to the PTAP motif (nucleotide positions 2152 to 2163) were placed in-frame in the p6* reading frame.

### Cell Culture and DNA Transfection

HeLa cells were maintained in Dulbecco’s modified Eagle’s medium (Sigma) supplemented with 10% fetal bovine serum. Transfection with DNA was carried out using Lipofectamine 2000 (Invitrogen).

### Purification of HIV Particles

Viral particles were purified by the standard procedures. At 2 days post-transfection, culture media were clarified, filtered, and centrifuged through 20% (wt/vol) sucrose cushions in an SW55 rotor (Beckman Coulter) at 100,000×g for 2 hr at 4°C. Viral pellets were resuspended in PBS.

### Membrane Flotation Centrifugation

At 2 days post-transfection, HeLa cells were harvested and resuspended in buffer containing 50 mM Tris (pH 7.5), 1 mM EDTA, 150 mM NaCl, 1 mM dithiothreitol, 1 mM phenylmethylsulfonyl fluoride, and 1 µg/ml pepstatin A. Following brief sonication, the cell lysates were clarified at 500×g for 7 min at 4°C. The supernatants were adjusted to 70% (wt/vol) sucrose, placed at the bottom of each tube, and overlaid with 65% and 10% (wt/vol) sucrose step gradients in PBS. Equilibrium flotation centrifugation was performed in an SW55 rotor at 100,000×g for 16 hr at 4°C. Fractions were collected from the bottom to the top of each tube.

### Western Blot Analysis

Protein samples were subjected to sodium dodecyl sulfate-polyacrylamide gel electrophoresis (SDS-PAGE) in 10% polyacrylamide gels and were transferred to polyvinylidene difluoride membrane. The membrane was incubated with anti-HIV-1 p24 mouse antibody [48] and subsequently with horseradish peroxidase-conjugated secondary antibodies (Cappel).

### Immunofluorescent Staining and Confocal Microscopy

HeLa cells were fixed with 3.7% paraformaldehyde in PBS for 30 min at room temperature and were permeabilized with 0.1% Triton X-100 for 10 min at room temperature. Following blocking with 1% bovine serum albumin in PBS, the cells were incubated with anti-HA mouse (Sigma), anti-FLAG rabbit (Sigma), or anti-HIV-1 p24 mouse [48] antibodies and subsequently with Alexa Fluor 488 or 568-conjugated antibodies (Molecular Probes). After nuclear staining with TO-PRO-3 (Molecular Probes), the cells were mounted with an antibleaching reagent and observed with a laser-scanning confocal microscope (Leica).

### Metabolic Labeling

At 27 hr post-transfection, cells were metabolically labeled with [^3^H]myristic acid (PerkinElmer) at 18.5 Mbq/ml for 3 hr. After labeling, the cells were collected and analyzed by SDS-PAGE followed by fluorography.

### Electron Microscopy

HeLa cells were fixed with 2.5% glutaraldehyde in 0.1 M cacodylate buffer (pH 7.4) for 1 hr at 4°C prior to treatment with 2% osmium tetroxide for 1 hr at 4°C. Ultrathin sections were stained with uranyl acetate and lead citrate and examined with an electron microscope (H-7500, Hitachi) at 80 kV.

## Results

### HIV-1 GagPol and its C-terminally Truncated Derivatives are Incapable of Viral Particle Production Despite Full N-terminal Myristoylation

**Figure 1:**
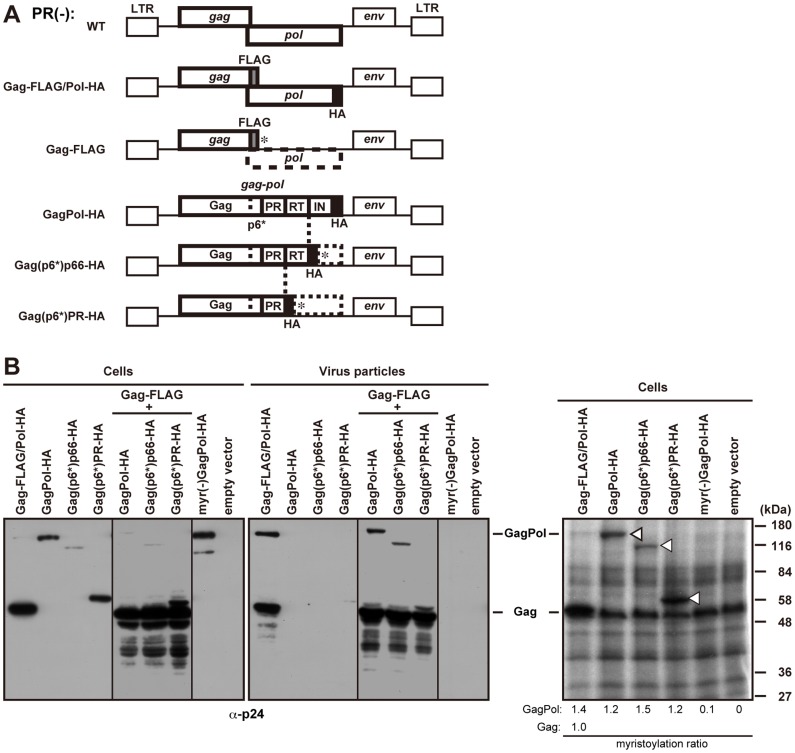
Viral particle production of HIV-1 GagPol and its C-terminally truncated derivatives. (A) Schematic representation of the pNL43 derivatives tagged with the FLAG and HA sequences. The pNL43 derivative containing inactive PR was used as wild type (WT). The FLAG and HA sequences were inserted in-frame in the C-terminal p6 domain of Gag and the C-terminus of GagPol, respectively (referred to as Gag-FLAG/Pol-HA) [44]. For expression of Gag-FLAG alone (without GagPol), the FLAG sequence was inserted in the C-terminal p6 domain of Gag and termination codons (asterisk) were placed in-frame in the *pol* frame. For expression of GagPol-HA (without Gag), the HA sequence was added to the C-terminus of the GagPol protein [44]. For the C-terminal truncations of GagPol, the HA sequence followed by a termination codon (asterisk) was inserted at the PR/RT or RT/IN junction of GagPol [referred to as Gag(p6*)p66-HA and Gag(p6*)PR-HA, respectively]. GagPol-HA and the derivatives contained the frameshift mutation, and all constructs contained inactive PR. LTR, long terminal repeat. (B) N-myristoylation and viral particle production of the truncated GagPol proteins. HeLa cells were singly transfected with the GagPol-HA and its C-terminally truncated constructs, or doubly transfected with a combination of the GagPol-HA and Gag-FLAG constructs at a Gag-to-GagPol DNA ratio of 1∶10. Total DNA amounts were normalized to 8 µg with pUC plasmid. Gag-FLAG/Pol-HA and myr(-)GagPol-HA containing the myristoylation (G2A) mutation were used as positive and negative controls, respectively. Cells were labeled with [^3^H]myristic acid for 3 hr and were subjected to SDS-PAGE followed by fluorography. Arrowheads indicate myristoylated GagPol-HA, Gag(p6*)p66-HA, and Gag(p6*)PR-HA, respectively. The cells and purified viral particles were subjected to Western blotting using anti-HIV-1 p24 antibody. The intensity of the band corresponding to each construct in fluorographed gels and Western blots was measured by ImageJ software. For each construct, the band intensity in fluorographed gels was divided by the band intensity in Western blots. The myristoylation ratio of Gag was set at 1.0, and the myristoylation ratios of the constructs relative to the ratio of Gag were calculated.

Previous studies have shown that expression of HIV-1 GagPol alone fails to produce viral particles [38], [39]. The C-terminal truncation of GagPol still did not restore particle production ability even with high-level expression by a baculovirus system [49]. Recently, Gould’s group has shown similar results in mammalian cell systems [45]. To examine the defect of HIV-1 GagPol in particle assembly, we used the PR-inactive version of pNL43 derivatives, in which the *gag* and *pol* frames were placed into the same reading frame by deleting the frameshifting signal. For positive controls, we used the pNL43 derivative expressing inactive PR without epitope tags (referred to as WT) and the pNL43 derivative expressing Gag tagged with the FLAG sequence and GagPol with the HA sequence (referred to as Gag-FLAG/Pol-HA) (Fig. 1A), both of which produced viral particles at similar levels [44]. The pNL43 derivatives with C-terminal truncation of the Pol region were made by placing a termination codon with an HA tag at each domain junction of the Pol region (Fig. 1A). HeLa cells were transfected with these GagPol constructs, and their particle production abilities were examined by Western blotting using anti-HIV-1 p24 antibody (Fig. 1B). The results confirmed that neither the full-length GagPol nor the truncated GagPol derivatives produced viral particles when expressed alone, although they were incorporated into viral particles when coexpressed with Gag-FLAG (Fig. 1B). It is well known that the production of viral particles by HIV-1 Gag protein is dependent on N-myristoylation [8], [9]. To investigate the myristoylation of GagPol, HeLa cells were transfected with these GagPol constructs and were metabolically labeled with [^3^H]myristic acid. When the [^3^H] signals were normalized to the signals by Western blotting, the full-length GagPol and truncated GagPol proteins were found to be fully myristoylated, indicating that the budding defects of the GagPol constructs are not due to inefficient N-myristoylation (Fig. 1B, right panel).

### C-terminal Truncation of GagPol Restored its Membrane Binding and Plasma Membrane Targeting

**Figure 2:**
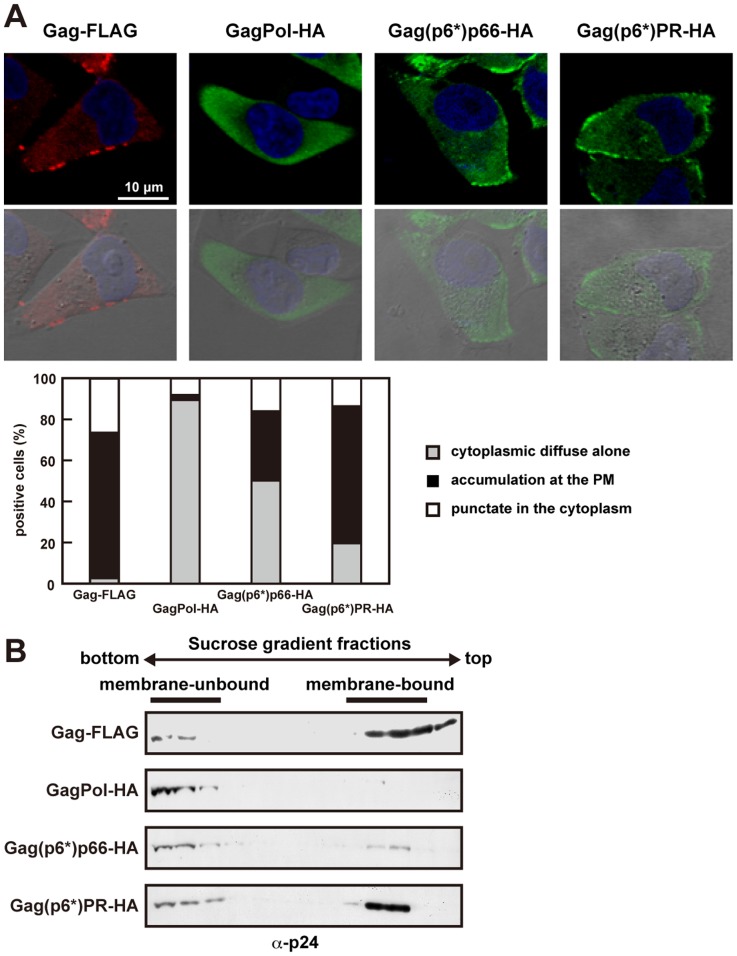
Membrane affinity and plasma membrane targeting of GagPol with C-terminal truncations. (A and B) HeLa cells were transfected with the Gag-FLAG, GagPol-HA, and C-terminally truncated constructs. Gag-FLAG was used as a positive control. (A) Intracellular localization of the truncated GagPol proteins. At 24 hr post-transfection, cells were immunostained with anti-FLAG (red) or anti-HA (green) antibodies and nuclei were stained with TO-PRO-3 (blue). Bottom panels show confocal images overlaid with differential interference contrast images. All micrographs are shown at the same magnification. In each sample, approximately 100 antigen-positive cells (from 3 or 4 independent experiments) were subjected to analysis of the antigen distribution pattern. (B) Membrane affinity of the truncated GagPol proteins. Cells were subjected to membrane flotation centrifugation followed by Western blotting using anti-p24 antibody. Representative blots in three independent experiments were shown.

To obtain clues to the cause of the defect, we observed GagPol-HA-transfected cells by confocal microscopy. The confocal images revealed only the diffuse distribution of GagPol-HA throughout the cytoplasm (Fig. 2A). Membrane flotation analysis revealed that GagPol-HA was incapable of binding to the membrane (Fig. 2B). When the intracellular localization of the C-terminally truncated derivatives was similarly analyzed by confocal microscopy, the antigen distribution at the plasma membrane became apparent with the progressive C-terminal truncation of GagPol (Fig. 2A). Consistent with these observations, membrane flotation analysis revealed the distribution of the GagPol derivatives to membrane-bound fractions, concomitant with the progressive truncation of GagPol (Fig. 2B). Since membrane targeting of Gag/GagPol is prerequisite for type C retrovirus particle assembly, we suggest that the membrane-binding defect of GagPol is primarily responsible for the defect of full-length GagPol in particle production. However, there was no correlation between particle production (Fig. 1) and membrane binding (Fig. 2) upon C-terminal truncations, suggesting that other stages (e.g., multimerization and particle budding) are potentially impaired.

### C-terminal Addition of Large Noncognate Protein to Gagp6*, Similar to the Pol Region, Reduced its Membrane Affinity

**Figure 3:**
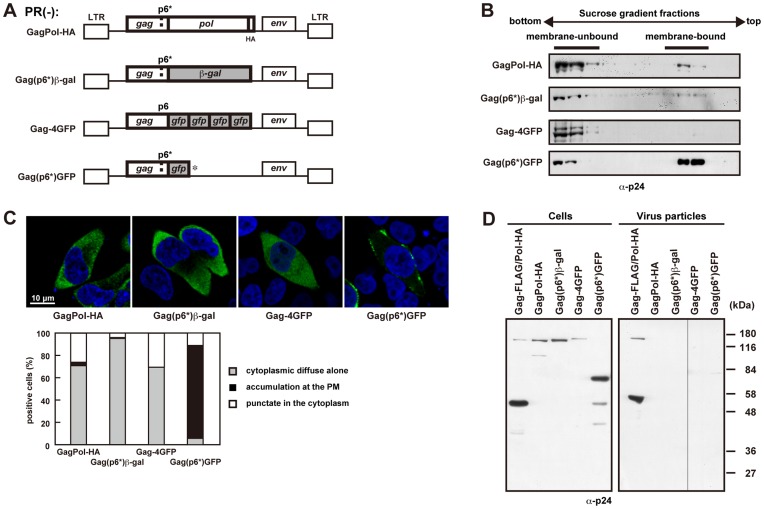
Membrane affinity and viral particle production of Gag with C-terminal extensions of noncognate proteins. (A) Schematic representation of the pNL43 derivatives in which the Pol region was replaced by noncognate proteins. The Pol region was replaced with ß-gal, GFP, and 4GFP [referred to as Gag(p6*)ß-gal, Gag(p6*)GFP, and Gag-4GFP, respectively]. The Gag(p6*)ß-gal and Gag(p6*)GFP contained the same frameshift mutation as described before. (B-D) HeLa cells were transfected with GagPol-HA, Gag(p6*)ß-gal, Gag(p6*)GFP, Gag-4GFP, and Gag-FLAG/Pol-HA. (B) Membrane affinity. At 24 hr post-transfection, cells were subjected to membrane flotation centrifugation followed by Western blotting using anti-p24 antibody. (C) Intracellular localization. The cells transfected with GagPol-HA or Gag(p6*)ß-gal were immunostained with anti-p24 antibody (green), and nuclei were stained with TO-PRO-3 (blue). All micrographs are shown at the same magnification. In each sample, approximately 100 antigen-positive cells (from 3 or 4 independent experiments) were subjected to analysis of their distribution patterns. (D) Intracellular expression and viral particle production. Cells and purified viral particles were subjected to Western blotting using anti-p24 antibody. Gag-FLAG/Pol-HA was used as a positive control.

To understand GagPol’s inhibitory effect on membrane binding, we replaced the Pol region with 120 kDa ß-gal and 27 kDa GFP [referred to as Gag(p6*)ß-gal and Gag(p6*)GFP, respectively]. We also made a Gag fusion construct that four tandem repeats of GFP, the length of which was nearly equivalent to the entire Pol region, were fused to the C-terminus of Gag (referred to as Gag-4GFP) (Fig. 3A). Membrane flotation analysis revealed the majorities of Gag(p6*)ß-gal and Gag-4GFP in non-membrane-bound fractions, similar to the case with GagPol-HA (Fig. 3B). In contrast, Gag(p6*)GFP was distributed to membrane-bound fractions at levels similar to those of Gag(p6*)PR-HA (compare with Fig. 2B). Confocal images were consistent with these findings, showing diffuse distribution of Gag(p6*)ß-gal and Gag-4GFP throughout the cytoplasm but, in contrast, Gag(p6*)GFP accumulation at the plasma membrane (Fig. 3C). These data suggest that the membrane-binding defect of GagPol was imposed by the C-terminal long extension, likely due to the length of the extension but not the specificity of the amino acid sequence. None of these constructs produced viral particles (Fig. 3D).

### The Budding Defect of Gag(p6*)PR is not Caused by its Lack of p6 Domain

**Figure 4:**
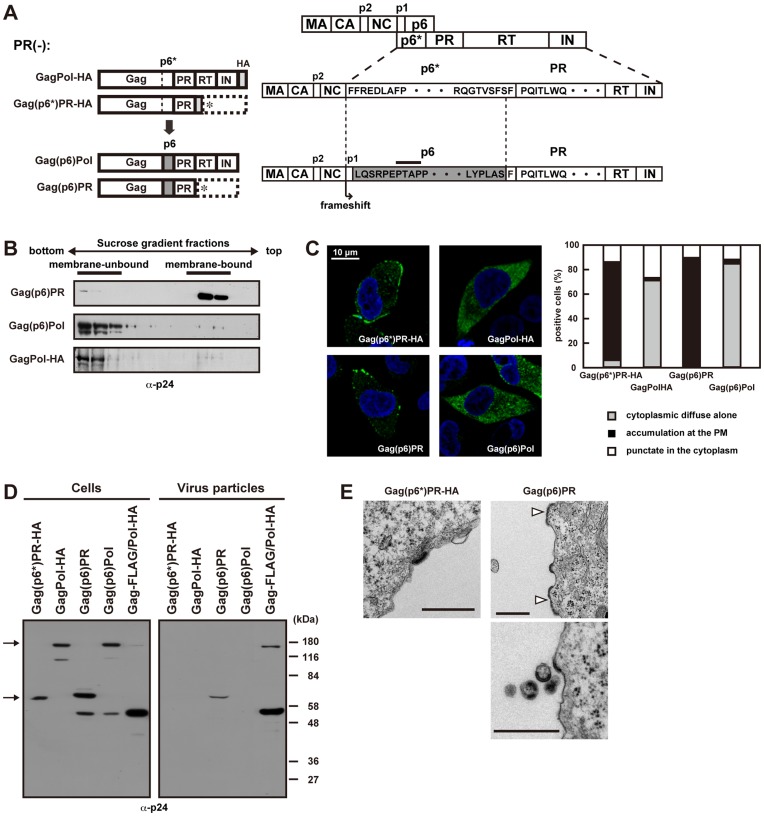
Viral particle production of GagPol constructs containing the p6 domain. (A) Schematic representation of the GagPol and GagPR constructs and their amino acid sequences of the p6* and p6 domains. The p6* domain was replaced by the p1+p6 domain (lacking the 12 C-terminal amino acids), and the resultant constructs were referred to as Gag(p6)Pol and Gag(p6)PR. The GagPol-HA and Gag(p6*)PR-HA constructs contain the authentic p6* domain (upper), and the Gag(p6)Pol and Gag(p6)PR constructs contain the p6 domain instead of the p6* (lower). All constructs contained inactive PR and were expressed in the context of pNL43. (B-E) HeLa cells were transfected with Gag(p6)Pol, Gag(p6)PR, GagPol-HA, and Gag(p6*)PR-HA constructs. (B) Membrane affinity of the Gag(p6)Pol and Gag(p6)PR proteins. Cells were subjected to membrane flotation centrifugation followed by Western blotting using anti-p24 antibody. (C) Intracellular localization of the Gag(p6)Pol and Gag(p6)PR proteins. Cells were immunostained with anti-p24 antibody (green), and nuclei were stained with TO-PRO-3 (blue). All micrographs are shown at the same magnification. In each sample, approximately 100 antigen-positive cells (from 3 independent experiments) were subjected to distribution pattern analysis. (D) Intracellular expression and viral particle production of the Gag(p6)Pol and Gag(p6)PR proteins. The Gag-FLAG/Pol-HA construct was used as a positive control. Cells and purified viral particles were subjected to Western blotting using anti-p24 antibody. Arrows indicate GagPol and GagPR. (E) Electron microscopy of cells transfected with Gag(p6*)PR-HA and Gag(p6)PR. The cells were stained with uranyl acetate and lead citrate. Arrowheads show pedestal-like structures. Bars, 500 nm.

It is well known that the deletion of the PTAP motif in the Gag p6 domain blocks the pinching off of viral particles at a late stage of budding [16], [18], [46], [50]. We reasoned that the budding defect of our constructs containing p6* might be partly due to a lack of the PTAP motif in the context of constructs, because the *gag*-to-*pol* frameshifting occurs upstream from the PTAP motif (Fig. 4A). Based on this hypothesis, we replaced the p6* with the p6 in the GagPol and Gag(p6*)PR constructs [referred to as Gag(p6)Pol and Gag(p6)PR, respectively] (Fig. 4A). As expected, membrane flotation analysis confirmed the membrane affinity of Gag(p6)PR but not that of Gag(p6)Pol (Fig. 4B). Confocal images showed antigen accumulation at the plasma membrane in Gag(p6)PR-expressing cells (Fig. 4C). However, Gag(p6)PR showed very little particle production (Fig. 4D), indicating that the p6 domain, most likely the PTAP motif, does not support efficient particle release in the case of Gag(p6)PR. A similar observation, that the budding defect of GagPol is not ascribable to the lack of p6, was reported in a very recent study [45]. These data suggested that the budding defect of Gag(p6*)PR is not due to the lack of p6 and raised the possibility that viral particle production by Gag(p6*)PR is blocked at other stages, such as Gag multimerization. We then used electron microscopy to examine this possibility (Fig. 4E). Cells expressing Gag(p6*)PR showed slightly curved, electron-dense structures at the plasma membrane but no spherical budding structures. Cells expressing Gag(p6)PR displayed aberrant morphology at the plasma membrane: some cells showed not spherical but pedestal-like electron-dense structures (5 out of 15 cells observed), distinctive from particles arrested at a late budding stage by PTAP deletion (Fig. 4E). Viral particles often displayed aberrant morphology carrying electron-dense materials (6 out of 15 cells observed). Such budding particles in an irregular shape by Gag(p6)PR have also been observed in insect cells [49]. These structures were not observed in untransfected cells. We speculated that the Gag construct ending at the PR domain failed to multimerize correctly at the plasma membrane during particle assembly. Alternatively, such Gag constructs may impair membrane curvature.

### GagPol Failed to Produce Viral Particles Even when Recruited to the Plasma Membrane

**Figure 5:**
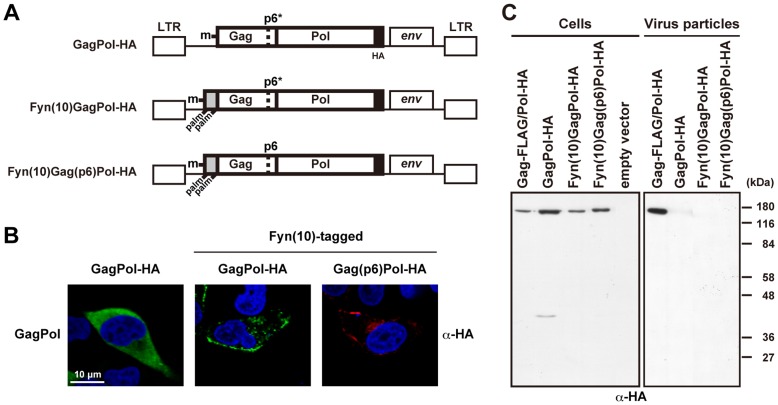
Plasma membrane targeting and viral particle production of GagPol with the Fyn(10) N-terminal sequence. (A) Schematic representation of GagPol-HA and its derivatives containing the Fyn(10) N-terminal sequence and the p6 domain. The initiation codon of GagPol-HA was replaced by Fyn(10) [referred to as Fyn(10)GagPol-HA] and the p6* domain was further replaced by the p6 domain [referred to as Fyn(10)Gag(p6)Pol-HA]. All constructs contained inactive PR. The letter **m** indicates a myristoylation site, and **palm** indicates a palmitoylation site. (B and C) HeLa cells were transfected with GagPol-HA, Fyn(10)GagPol-HA, and Fyn(10)Gag(p6)Pol-HA. (B) Intracellular localization of GagPol-HA derivatives. Cells were immunostained with anti-HA antibody (green or red) and nuclei were stained with TO-PRO-3 (blue). All micrographs are shown at the same magnification. (C) Intracellular expression and viral particle production. The Gag-FLAG/Pol-HA construct was used as a positive control. Cells and purified viral particles were subjected to Western blotting using anti-HA antibody.

In our study, despite N-myristoylation, GagPol protein was incapable of binding to the membrane. This raised the possibility that the presence of an N-myristoyl moiety alone was insufficient for membrane targeting in the context of GagPol. To resolve this issue, we constructed GagPol derivatives, whose initiation codons were replaced by Fyn(10) [47], [51], a tight membrane-binding signal containing one myristoylation and two palmitoylation sites (Fig. 5A). The addition of the Fyn(10) signal to Gag has been shown to rescue the membrane-binding defect imposed by depletion of PI(4,5)P_2_, a host factor that triggers the exposure of the myristoyl moiety [11], [52], [53]. Confocal images revealed that expression of the authentic GagPol alone showed only diffuse cytoplasmic staining. In contrast, Fyn(10)GagPol constructs were accumulated at the plasma membrane (Fig. 5B). However, Fyn(10)GagPol-HA still did not produce viral particles (Fig. 5C), and the budding defect of Fyn(10)GagPol-HA was not rescued by replacing the p6* domain with the p6 domain [referred to as Fyn(10)Gag(p6)Pol-HA] (Fig. 5C). Altogether, our data indicate that large C-terminal extension to Gag, such as the Pol region, imposes the virion release defects primarily in membrane binding and potentially in assembly.

## Discussion

It has long been known that GagPol alone is incapable of viral particle production and is incorporated into viral particles only by coassembly with Gag [38], [39]. Some studies on C-terminal truncation and replacement of p6* with p6 have suggested that the defect of GagPol in particle budding was not due to the lack of p6 but to the presence of the Pol region [39], [45], [49]. In the present study, we demonstrated that the budding defect of GagPol was ascribable to the lack of membrane affinity despite full N-myristoylation.

The membrane-binding ability of Gag lies within the N-terminal MA domain that contains N-myristoylation and a cluster of basic residues, although multimerization of the CA domain has been shown to enhance the membrane binding of Gag, possibly due to the exposure of the N-terminal myristoyl moiety [5], [13]. The N-terminal half of GagPol is nearly identical to Gag (MA-CA-NC), indicating that GagPol harbors all the elements supportive of its membrane binding. Nonetheless, GagPol fails to bind to the membrane. Our data, together with the previous findings, suggest the following possible explanations for the membrane-binding defect of GagPol, although alternatives cannot be ruled out. (i) The N-terminal myristoyl moiety may not be exposed in the context of GagPol. Recent NMR studies of MA have indicated that the N-terminal myristoyl moiety becomes exposed upon PI(4,5)P_2_ binding to the basic amino acids of MA [11]. If GagPol fails to bind to PI(4,5)P_2_, then the myristoyl moiety of GagPol may similarly remain to be occluded. Although liposome-binding assays do not allow us to provide such evidence because of limitation of GagPol detection, we found that the replacement of the authentic myristoyl signal by the Fyn(10) signal, which has been shown to rescue the Gag membrane-binding defect imposed by PI(4,5)P_2_ depletion [53], conferred membrane-binding ability to GagPol (Fig. 5B). (ii) Alternatively, even if the myristoyl moiety is exposed, it and PI(4,5)P_2_ binding might not suffice for stable binding to the membrane in the case of GagPol, and this would result in the rapid dissociation of GagPol from the membrane. One study has suggested that the myristoylated glycine confers 8 kcal/mol to membrane binding [54]. Recent theoretical calculations of electrostatic interactions and liposome-binding assays have indicated that monomeric HIV-1 MA has a membrane-binding energy of 5 kcal/mol [electrostatics, increasing to 7.5 kcal/mol when the membrane contains 1% PI(4,5)P_2_] and 4 kcal/mol (myristoyl moiety, if exposed) [55]–[57]. This total binding energy suffices for membrane binding of Gag but may not of GagPol, since the tight membrane-binding signal Fyn(10) (containing one myristoyl and two palmitoyl moieties) was required for the membrane binding of GagPol.

Our truncation experiments indicated that the membrane affinity of GagPol was recovered upon progressive C-terminal truncation of the Pol region (Fig. 2B). The truncated constructs showed no particle production [in the case of Gag(p6*)p66-HA] or very little particle production [in the case of Gag(p6*)PR-HA] (Fig. 1B). Similar observations, e.g., the more progressive the Pol truncation, the less defective Pol is for particle formation, have been reported, although the extent of particle production by GagPR varied in different cell systems (293T and insect cells) [45], [49]. These studies also indicated that GagPRRT failed to produce viral particles by electron microscopic analysis [49] and by Western blotting of the particle fractions [45]. The latter study suggested that the RT region was responsible for the defect of GagPol particle budding. Our study made similar observations but revealed by membrane flotation analysis that the budding defects were linked with no or little membrane-binding ability in the case of Gag(p6*)p66 and GagPol (Fig. 2B). More importantly, our substitution experiments clearly show that membrane binding is also impaired if the Pol region is replaced by a noncognate large protein (e.g., ß-gal, 4GFP) (Fig. 3B). Although we cannot exclude a possibility that ß-gal and 4GFP may aggregate as dimers or oligomers and impair the membrane-binding ability, at least, our data indicate that the budding defect observed for GagPol is not caused by the RT sequence. It remains to be elucidated why C-terminal long extensions to Gag impair the membrane binding of Gag. Recent studies have suggested that the basic clusters present in the MA and NC domains both bind to RNA and fold Gag into a compact structure [58], [59] but that the RNA on the MA basic cluster gets displaced by PI(4,5)P_2_ enriched at the plasma membrane [60], [61], possibly allowing Gag to stretch out to stimulate its multimerization. Although little is known about the GagPol-RNA-membrane interactions, one report has shown that RNA does not bind to the NC domain within GagPol but facilitates Gag-GagPol interactions [62]. These studies suggest a possibility that the RNA bound to Gag also binds to the MA domain of GagPol. It is tempting to speculate that GagPol alone lacks the membrane-binding ability because its MA domain is masked by RNA but the RNA-mediated Gag-GagPol interactions can bring GagPol to the plasma membrane.

It is well known that the deletion of the PTAP motif or the p6 domain from Gag arrests particle budding at a late stage, showing viral particles tethered to the plasma membrane. In our study, however, even though Gag(p6*)PR-HA accumulated at the plasma membrane, it did not show particle-like structures. The Gag(p6)PR construct, despite having the p6 domain, did not produce spherical budding particles (Fig. 4E). These data raise the possibility that the PR domain at least in the context of GagPR might inhibit correct assembly and/or membrane curvature at an early step of particle budding. The aberration of particle assembly by the presence of C-terminal PR has been observed for HIV-1 in insect cells [49]. Rous sarcoma virus Gag protein naturally includes the PR domain at its C-terminus and produces virus particles in avian and mammalian cells, but insect cells it showed virion assembly defect, which was rescued by deletion of the PR domain [63]. Although we still do not understand exactly how HIV-1 Pol impairs Gag membrane binding or how PR impairs Gag assembly, our observations in this study provide important clues toward a full understanding of the budding defect of retroviral GagPol.
