# Correction: A Large Extension to HIV-1 Gag, Like Pol, Has Negative Impacts on Virion Assembly

**DOI:** 10.1371/journal.pone.0117765

**Published:** 2015-01-21

**Authors:** The PLOS ONE Staff

There are errors in the Western blot panels of Fig. 4D and Fig. 5C. Please view the correct figures below.

**Fig 4:**
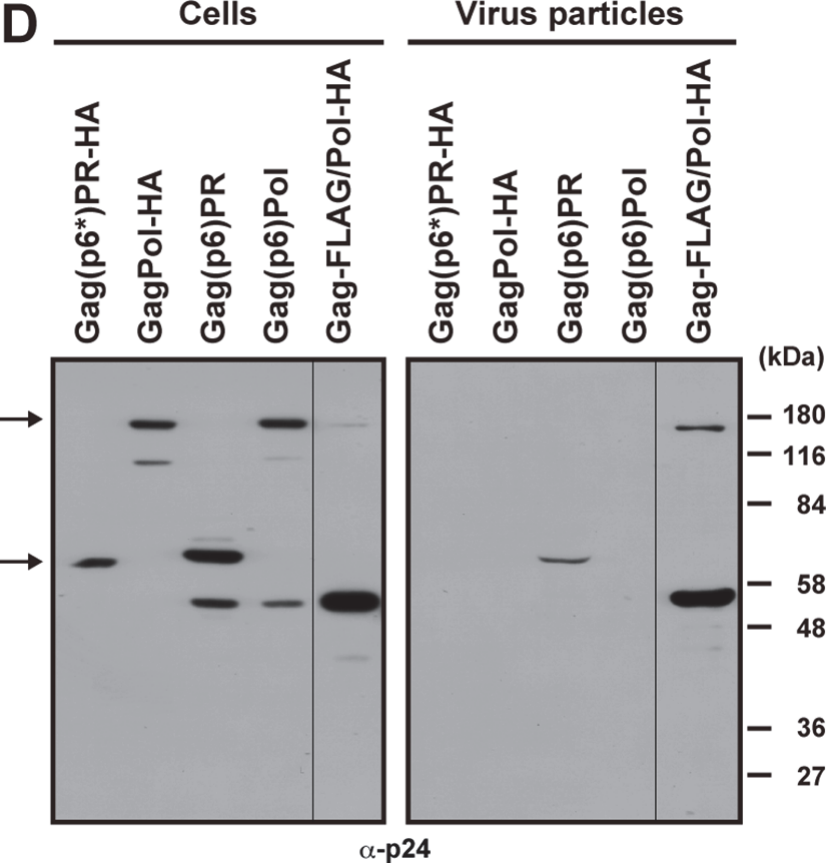
Viral particle production of GagPol constructs containing the p6 domain. (A) Schematic representation of the GagPol and GagPR constructs and their amino acid sequences of the p6* and p6 domains. The p6* domain was replaced by the p1+p6 domain (lacking the 12 C-terminal amino acids), and the resultant constructs were referred to as Gag(p6)Pol and Gag(p6)PR. The GagPol-HA and Gag(p6*)PR-HA constructs contain the authentic p6* domain (upper), and the Gag(p6)Pol and Gag(p6)PR constructs contain the p6 domain instead of the p6* (lower). All constructs contained inactive PR and were expressed in the context of pNL43. (B-E) HeLa cells were transfected with Gag(p6)Pol, Gag(p6)PR, GagPol-HA, and Gag(p6*)PR-HA constructs. (B) Membrane affinity of the Gag(p6)Pol and Gag(p6)PR proteins. Cells were subjected to membrane flotation centrifugation followed by Western blotting using anti-p24 antibody. (C) Intracellular localization of the Gag(p6)Pol and Gag(p6)PR proteins. Cells were immunostained with anti-p24 antibody (green), and nuclei were stained with TO-PRO-3 (blue). All micrographs are shown at the same magnification. In each sample, approximately 100 antigen-positive cells (from 3 independent experiments) were subjected to distribution pattern analysis. (D) Intracellular expression and viral particle production of the Gag(p6)Pol and Gag(p6)PR proteins. The Gag-FLAG/Pol-HA construct was used as a positive control. Cells and purified viral particles were subjected to Western blotting using anti-p24 antibody. Arrows indicate GagPol and GagPR. (E) Electron microscopy of cells transfected with Gag(p6*)PR-HA and Gag(p6)PR. The cells were stained with uranyl acetate and lead citrate. Arrowheads show pedestal-like structures. Bars, 500 nm.

**Fig 5:**
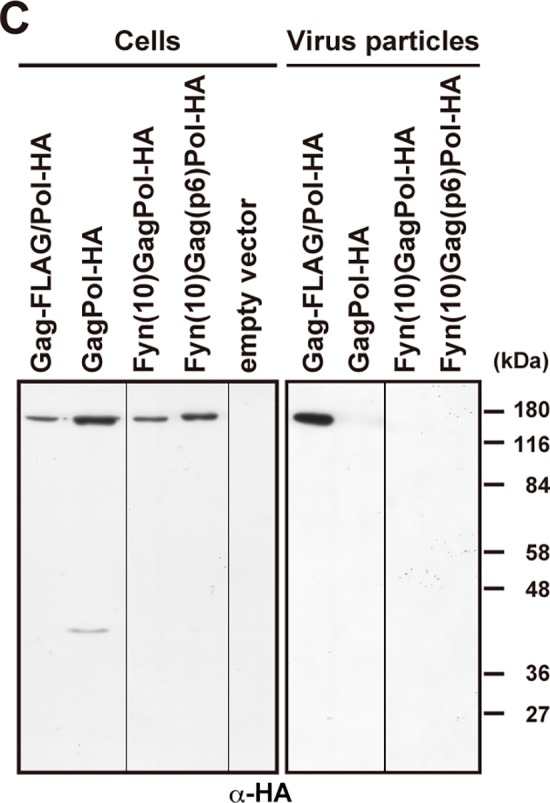
Plasma membrane targeting and viral particle production of GagPol with the Fyn(10) N-terminal sequence. (A) Schematic representation of GagPol-HA and its derivatives containing the Fyn(10) N-terminal sequence and the p6 domain. The initiation codon of GagPol-HA was replaced by Fyn(10) [referred to as Fyn(10)GagPol-HA] and the p6* domain was further replaced by the p6 domain [referred to as Fyn(10)Gag(p6)Pol-HA]. All constructs contained inactive PR. The letter **m** indicates a myristoylation site, and **palm** indicates a palmitoylation site. (B and C) HeLa cells were transfected with GagPol-HA, Fyn(10)GagPol-HA, and Fyn(10)Gag(p6)Pol-HA. (B) Intracellular localization of GagPol-HA derivatives. Cells were immunostained with anti-HA antibody (green or red) and nuclei were stained with TO-PRO-3 (blue). All micrographs are shown at the same magnification. (C) Intracellular expression and viral particle production. The Gag-FLAG/Pol-HA construct was used as a positive control. Cells and purified viral particles were subjected to Western blotting using anti-HA antibody.
